# High Survivorship of First-Generation Monarch Butterfly Eggs to Third Instar Associated with a Diverse Arthropod Community

**DOI:** 10.3390/insects12060567

**Published:** 2021-06-21

**Authors:** Misty Stevenson, Kalynn L. Hudman, Alyx Scott, Kelsey Contreras, Jeffrey G. Kopachena

**Affiliations:** 1 Dallas Arboretum and Botanical Garden, 8525 Garland Road, Dallas, TX 75218, USA; mnixon@dallasarboretum.org; 2 Department of Biological and Environmental Sciences, Texas AM University—Commerce, Commerce, TX 75428, USA; kalynnhudman@gmail.com; 3 Houston Zoo, 6200 Herman Park Drive, Houston, TX 77030, USA; ascott@houstonzoo.org; 4 Environmental Health and Safety, University of Texas at Arlington, Arlington, TX 76019, USA; Kelsey.Contreras@uta.edu

**Keywords:** monarch butterfly, *Danaus plexippus*, arthropods, community structure, survivorship

## Abstract

**Simple Summary:**

The eastern migratory population of the monarch butterfly has been the focus of extensive conservation efforts in recent years. However, there are gaps in our knowledge about the survival of first, or spring generation, monarchs in their core areas of Texas, Oklahoma, and Louisiana. This is important because the spring generation represents the first stage of annual recovery from overwinter mortality. It is, therefore, an important stage for monarch conservation efforts. This study showed that, in the context of a complex arthropod community in north Texas, first generation monarch survival was high. The study found that survival was not directly related to predators on the host plant, but was higher on host plants that harbored a greater number and variety of other, non-predatory arthropods. This is possibly because the presence of alternate, preferable prey enabled monarch eggs and larvae to be overlooked by predators. The implication is that, at least in the southern U.S., monarch conservation should consider strategies that promote diverse functional arthropod communities.

**Abstract:**

Based on surveys of winter roost sites, the eastern migratory population of the monarch butterfly (*Danaus plexippus*) in North America appears to have declined in the last 20 years and this has prompted the implementation of numerous conservation strategies. However, there is little information on the survivorship of first-generation monarchs in the core area of occupancy in Texas, Oklahoma, and Louisiana where overwinter population recovery begins. The purpose of this study was to determine the survivorship of first-generation eggs to third instars at a site in north Texas and to evaluate host plant arthropods for their effect on survivorship. Survivorship to third instar averaged 13.4% and varied from 11.7% to 15.6% over three years. The host plants harbored 77 arthropod taxa, including 27 predatory taxa. Despite their abundance, neither predator abundance nor predator richness predicted monarch survival. However, host plants upon which monarchs survived often harbored higher numbers of non-predatory arthropod taxa and more individuals of non-predatory taxa. These results suggest that ecological processes may have buffered the effects of predators and improved monarch survival in our study. The creation of diverse functional arthropod communities should be considered for effective monarch conservation, particularly in southern latitudes.

## 1. Introduction

The monarch butterfly (*Danaus plexippus*) is an iconic North American butterfly whose seasonal distribution spans much of North America [1,2,3,4,5]. However, despite this large geographic distribution, based on censuses of overwintering sites there have been marked declines in populations of this species. The eastern migratory population, which occurs in much of North America east of the Rocky Mountains [2], has shown a decline of over 80% in the last 20 years at overwintering sites in Mexico [6,7]. In response to this rapid decline, the eastern migratory population of the monarch butterfly was petitioned for listing under the Endangered Species Act (ESA) in 2014 [3,8]. In the fall of 2020, the U.S. Fish and Wildlife Service (USFWS) ruled that listing the monarch butterfly under the ESA was warranted, but was precluded because limited resources had to be expended on higher-priority species [3]. The issue of monarch population declines is a complex one. However, despite some contrasting perspectives [9,10,11] the development of an interagency Monarch Joint Venture [12] and extensive publicity has resulted in the expenditure of millions of dollars and the investment of considerable labor toward conservation activities designed to increase monarch populations. A study conducted in 2014 [13] indicated that the U.S. public is willing to spend between $4.78 and $6.64 billion dollars on monarch conservation. Clearly, such a high potential expenditure requires informative data to ensure that conservation activities are most effectively implemented.

The eastern migratory population of the monarch butterfly colonizes North America each spring and summer through a series of four or five generations [2]. With the possible exception of a small population that may winter among Caribbean islands [14], the entire eastern population of monarch butterflies spend the winter in a few roosting sites in central Mexico [15]. Population size reaches its minimum in the early spring after overwinter mortality and most of these surviving individuals migrate north to lay eggs in a relatively small geographic area in Oklahoma, Texas, and western Louisiana [2,16,17]. The eggs laid by these migrants represent the first, or spring generation of the eastern population. Subsequent generations and the resulting expansion of the population through eastern North America depends on recruitment from this first generation. For this reason, productivity of first-generation monarchs in the southern U.S. has been cited as an important area for conservation efforts [18,19,20,21]. Because of this, there is a critical need for data on the survival of first-generation monarchs in order for appropriate conservation strategies to be developed [22].

Despite the fact that the first generation appears to be an important bottleneck in the annual growth of eastern monarch populations [20,21], there is almost no information on the ecology and success of this generation. This gap in knowledge creates uncertainty in what measures, if any, need to be taken to increase the fecundity and survival of this generation [21]. There are only three studies that measure the survivorship of first-generation monarchs in the core areas of Texas, Oklahoma and western Louisiana [23,24,25]. The most recent of these studies [25] was over 20 years ago, and none of the three studies provide comprehensive data on the ecological context associated with survivorship. An important purpose of the current study was to provide updated information on first generation survival in this region and to provide details on the ecological context of this generation. 

The three studies cited above all implicate arthropod predation as important factors limiting the survival of first-generation monarchs. However, these studies do not provide details on the arthropod communities associated with the host plants. In other geographic areas, and for other generations, arthropods are important correlates of monarch egg and larval mortality [26,27,28,29,30,31,32,33,34,35,36] and monarch eggs and larvae are subject to an extraordinary variety of arthropod predators [30,34]. Most of these studies quantify monarch mortality by looking at rates of loss to specific predators under very controlled experimental conditions. Very few studies examine survival in the context of the natural host plant arthropod community which includes non-predatory species as well as predatory species. Among the few studies that do look at community-level interactions, there is considerable variation in how host-plant arthropods affect monarch survival. In some cases, survival is higher in simple, species-poor communities, than it is in more complex communities [26,35,37]. In other cases, survival is higher in more complex arthropod communities than in less complex communities [28,38], possibly due to indirect top-down effects [27]. 

Understanding the ecological context of monarch survival is important because a major component of the Monarch Conservation Implementation Plan [12], prepared by the Monarch Joint Venture, is to plant more milkweed plants throughout the species’ breeding distribution. In response considerable effort has been made in planting milkweed plants in a variety of settings, including urban monarch gardens, in order to increase the availability of milkweed host plants [39,40]. However, simply planting milkweed plants ignores the potential importance that arthropod community interactions might have on monarch survival. The creation of these anthropogenic environments could, in fact, have the opposite effect by creating ecological traps [41,42].

Here we present an in-depth, up-to-date assessment of first-generation monarch survival across three years at a study site in northeast Texas. Our goal was to quantify egg and larval survival, to document the arthropod community associated with monarch butterfly host plants, and to evaluate how host-plant arthropods impact monarch survival. 

## 2. Materials and Methods

Data on monarch egg and larval survival were collected at the Cooper Wildlife Management Area and adjacent portions of Cooper Lake State Park in Hopkins Co., TX, USA, (33°18′51.09″ N, 95°36′16.70″ W) during the springs of 2016 through 2018. In 2016, data were collected from 28 March through 14 May, in 2017, data were collected from 21 March through 17 May, and in 2018, data were collected from 26 March through 11 May. The onset of each field season occurred when the first adults arrived and ended when eggs could no longer be found and all eggs had either reached the third instar or perished. The 2016 field study was a pilot project and, in that year, the only data that was collected was survival of eggs and larvae. More thorough studies were conducted in 2017 and 2018. 

The study area contained 48 ha of old-field habitat with isolated stands of trees and woodland edges. The vegetation consisted of a diverse mixture of native and exotic grasses and forbs. The only species of milkweed present was *Asclepias viridis* and its density, measured in 2017 using thirty 50 m^2^ circular plots, was 6540 plants per ha., or about 17,015 ramets per ha. 

We found Monarch eggs by either by watching females oviposit or by searching individual milkweed plants. Once an egg was found, the plant was marked with a flag and the leaf containing the egg was marked with a non-toxic marker. We followed the focal animal sampling methods used by Prysby [30] and by De Anda and Oberhauser [33] to monitor each egg daily, between 10:00 h and 17:00 h, from the day it was found until it reached the third instar or the egg or larva was missing from the plant. As in other studies that used focal samples, we considered a larva to be dead if was missing from the plant [43,44]. However, early instar larvae can be difficult to find on the host plant and monarch larvae at all stages are known to temporarily leave the host plant for a variety of reasons [45,46]. Therefore, to ensure that a larva had not been overlooked or was temporarily off the host plant, we continued to monitor the plant for four days after a larva was missing from the plant. If the larva was not detected during those four days, it was considered dead and the date of its mortality was recorded as the day it was first missing from the host plant. Furthermore, during our pilot study in 2016, when some host plants were enclosed to exclude predators (data not reported here), it was found that after the larvae reached the third instar, they began to emigrate off the host plant. The tendency to leave the host plant at or after the third instar has also been observed in other studies [33,47]. This meant that once the larvae reached the third instar, we could not distinguish between emigration and mortality. For that reason, we measured survival only up to the third instar. 

In 2017 and 2018, data were collected on all other arthropods found on the host plants. To do so, each host plant was approached carefully and all arthropods on the plant were observed and recorded during this approach. Other, less mobile, arthropods were recorded upon close examination of the plant and during the course of searching for the egg or larva. This approach clearly has limitations. In order to avoid disturbing the community, none of the arthropods could be collected, whereas other arthropods would leave the host plant upon approach. As a result, though we tried to be as specific as possible, it was not possible to identify many arthropods beyond the family level. Furthermore, it is acknowledged that these observations represent only a snapshot of the arthropod community on the host plant at a given moment in time. Our interpretations of this data are made with these limitations in mind. 

We also measured aspects of the size of the host plants. On the first and last days of monitoring a host plant, we measured the number of ramets, the length of each ramet, and the number of mature leaves on each ramet. For the purpose of analyses, we took the average of the two sets of measurements to quantify host plant size parameters. 

Statistical analyses were conducted using SAS^®^ Studio 3.8 software. In reporting the results of statistical tests, we focused on effect sizes. However, we used *p*-values of ≤0.05 to indicate effect sizes that were different from each other or from random values [48]. The simple analysis of survival to the third instar was based on frequency data. To compare survival among years, we used a simple chi-square contingency table analysis. To analyze survival relative to the arthropod community we used logistic regression to test which groups of arthropods best predicted monarch survival. In this case, a stepwise variable selection procedure was used to generate a subset of predictive models. We then used corrected Akaike’s information criteria scores (AICc) to select the model with the best fit (lowest AICc) [49] from among the candidate models. We followed this analysis with a more general comparison of the abundance and richness of predatory and non-predatory arthropods associated with eggs that survived and those that did not survive. Since these data were not normally distributed Kruskal–Wallis tests were used and *p*-values of 0.05 were used to distinguish effect sizes that were significantly different from random. 

## 3. Results 

### 3.1. Survival of Monarch Eggs and Larvae

**Figure 1:**
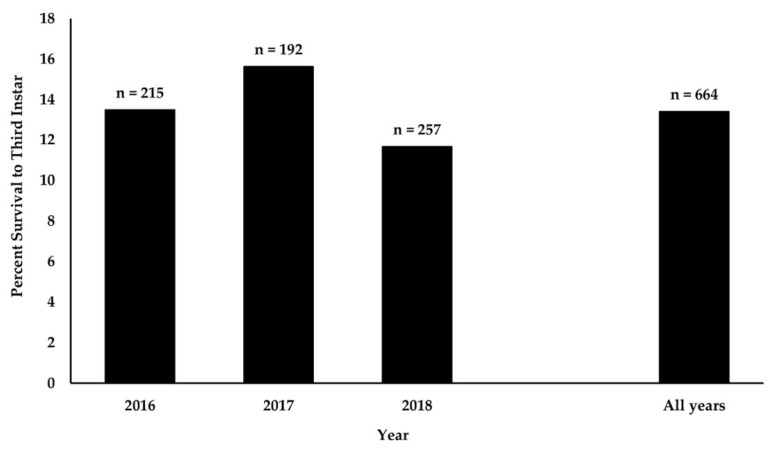
Percent survival of monarch eggs to third instar for each year of the study and for all years combined. Statistical analyses show that differences among years were minor. Chi-square test, 2 × 3 contingency table, Chi-square = 1.48, df = 2, *p* = 0.477. Numbers over bars indicate the number of eggs included in the analysis.

The survivorship of monarch butterfly eggs to the third instar was monitored for 664 eggs on 401 host plants; 215 eggs in 2016, 192 eggs in 2017, and 257 eggs in 2018. Survivorship was rather consistent among years and varied from 11.7% in 2018 to 15.6% in 2017 (Figure 1). The overall survivorship from egg to third instar for all three years combined was 13.4%.

### 3.2. Host Plant Arthropods and the Survival of Monarch Eggs and Larvae

Our primary focus for this analysis was to see if we could make inferences about unknown sources of mortality based on host plant arthropods. Our general observations in 2016 indicated that some host plants consistently harbored more arthropods than others. We wanted to know how this variation in arthropod activity affected monarch egg and larval survival. In 2017 and 2018, data on host plant arthropods were collected for 449 eggs; 192 eggs in 2017 and 257 eggs in 2018. Of these eggs and subsequent larvae, 42 died because the plants were either trampled by wildlife, had severe stem damage from wind, or were browsed by rabbits [50]. This source of mortality represented 3% of all mortalities in 2017, 16% of all mortalities in 2018, and 11% of all mortalities for both years combined. For the purposes of running a logistic regression analysis of survival based on arthropod groups, we were interested only in making inferences about unknown sources of mortality. Because mortality caused by plant damage or browsing was a known source of mortality, these individuals were eliminated from the logistic regression analyses of the effects of arthropods on egg and larval survival.

Some eggs were infected by parasitic wasps (Hymenoptera, Apocrita, *Trichogramma*). In 2017 there were 13 parasitized eggs, accounting for 8% of overall mortality whereas in 2018 there were 5 parasitized eggs, accounting for 2% of all mortalities. Overall, *Trichogramma* parasitism was responsible for 5% of the mortalities recorded in this study. Since the source of the *Trichogramma* mortalities was known, and since our logistic regression analyses was focused on unknown sources of mortality, these eggs were also removed from the logistic regression analyses.

Lastly, we had to correct the data for the inherent bias associated with arthropod counts on host plants on which eggs survived and host plants upon which larvae died. The longer an egg or larva was monitored, the more likely it was that more kinds and greater numbers of arthropods would be associated with that individual. Since eggs that survived were often monitored for a longer period of time than eggs that died, these data would be biased in favor of detecting more arthropods associated with surviving monarch eggs and larvae. To eliminate this bias, 164 individuals that were monitored for less than 10 days were removed from the analysis. This resulted in data on 224 eggs on 174 host plants where the mean number of days monitored and the variance in the number of days monitored was essentially the same for eggs that survived (n = 164) and eggs that died (n = 60) (*t*-test for mean number of days, *t* = 0.16, *p* = 0.8765; Test for equal variances, F = 1.35, *p* = 0.1421). 

We documented 15,441 arthropods distributed among 77 different taxa on the host plants used in this analysis (Appendix A). This did not include the monarch eggs and larvae themselves. Of the 77 taxa, 27 were predatory, and three of the four most abundant taxa were predators. Six taxa were milkweed-feeding herbivores. The remaining 44 taxa were visiting the plants for nectar, harboring on the plants, or transients (Appendix A). 

Though aphids were the most abundant arthropods, they were not the most frequent. Over half of the host plants had jumping spiders on them (Appendix A). Other predators that showed a high frequency on host plants were little black ants (33%) and fire ants (31%). The most frequent non-predatory arthropods were aphids (36%), leafhoppers (34%) and unknown flies (32%). However, most arthropods were uncommon and 59 of the 77 taxa (77%) occurred on less than 10% of the host plants (Appendix A). 

**Figure 2:**
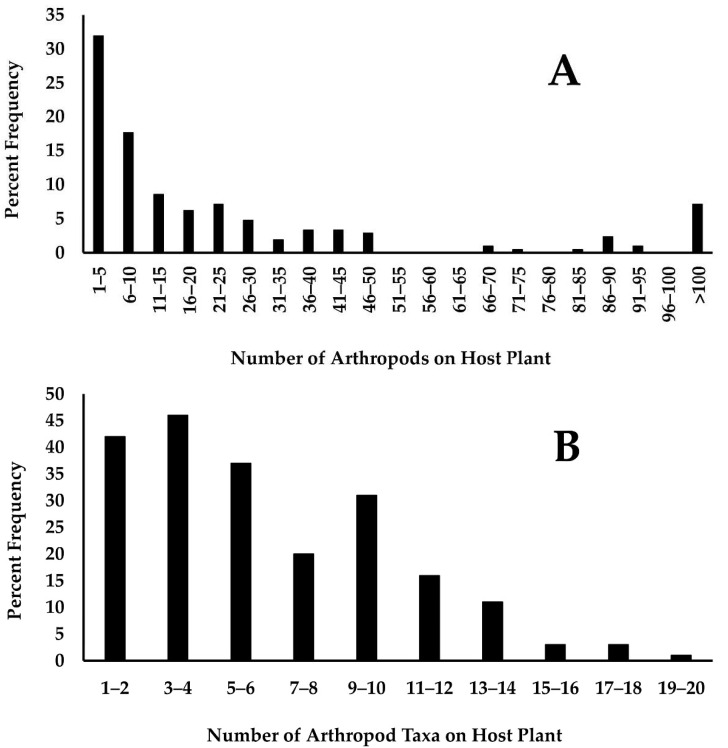
The number of arthropods and the number of arthropod taxa found on monarch host plants. (**A**) Total number of arthropods on monarch host plants grouped into intervals of five. (**B**) The number of taxa (taxon richness) found on monarch host plants grouped into intervals of two.

The low frequency of many of the arthropod taxa indicated that there was considerable variation among host plants. The total number of arthropods on a host plant was highly skewed, ranging from one through 4008 (Figure 2A). As a result, though the mean number of arthropods on a host plant was 73.5, the median number was 11 and most plants held only three arthropods. Similarly, as might be expected, the taxon richness of host plant arthropods was also highly skewed and ranged from one through 20 (Figure 2B). In this case the average richness was 6.2 and varied from 1 through 20 taxa. The median richness was five, but most host plants held four or fewer arthropod taxa. 

The low frequency of most arthropods made the data sparse and over-dispersed. While logistic regression is robust against deviations from normality [51], sparse data can lead to inflated parameter estimates and parameters with confidence intervals that approach infinity [52]. To avoid this issue, the arthropod taxa were combined into 16 groups based on frequency, food habits (predatory or non-predatory), taxonomic affiliation, and ecological similarity (Table 1). We used logistic regression to see if these arthropod groups predicted monarch egg and larval survival. In addition, since survival varied relative to date and since the number of arthropods is likely a product of plant size, we also included date, the number of ramets on the host plant, the total length of ramets on the host plant, and the total number of leaves of the host plant as candidate variables for the stepwise variable selection procedure used to identify the best predictive models. 

The results of this analysis did not identify any specific type of arthropod as having a large impact on monarch survival (Table 2). None of the predatory taxa or groups were included in any of the models, and the most important three groups were “Other Non-predatory Arthropods” “Mites” and “Other Ants”, of which only “Other Non-predatory Arthropods” significantly predicted monarch survival. Monarch survival was highest on plants that held a larger number of “Other Non-predatory Arthropods” (Table 2). Kruskal–Wallis tests corroborated this association between survival and non-predatory arthropods. When all of the non-predatory arthropods were combined into a single group, it was found that plants upon which eggs survived to the third instar generally had more non-predatory arthropods than did plants upon which eggs did not survive to the third instar (Survived: median = 10, mode = 0, range = 1258; died: median = 4, mode = 0, range = 3918; Kruskal–Wallis Chi-square = 6.40, *p* = 0.0114). When all predatory arthropods were combined, no important difference in predator abundance was found between plants where eggs survived and plants where they did not survive (survived: median = 4, mode = 1, range = 815; died: median = 2, mode = 1, range = 131; Kruskal–Wallis Chi-square = 2.41, *p* = 0.1202).

We wanted to know if these results could be generalized to predict monarch survival simply on the abundance and richness of predatory arthropods and non-predatory arthropods. For this purpose, we ran the logistic regression using only four variables: the number of predatory arthropods, the number of non-predatory arthropods, the taxon richness of predatory arthropods, and the taxon richness of non-predatory arthropods. Using these four variables, the stepwise procedure identified only two potential models as predictors of monarch survival (Table 3). In this case, the best model identified the taxon richness of non-predatory arthropods as a positive predictor of monarch survival. Interestingly, the two models selected by our procedure did not differ substantially in AIC weight (*w_i_*) and the second model indicated a positive relationship between the total number of predatory arthropods and monarch survival. However, the magnitude of that effect was extremely small (MLE = 0.00359) and not statistically significant (*p* = 0.2619). Kruskal–Wallis tests corroborated the positive association between egg survival and the number of non-predatory arthropod taxa. Plants upon which eggs survived to the third instar generally had a greater number of non-predatory arthropod taxa than did plants upon which eggs did not survive to the third instar (survived: median = 4, mode = 3, range = 14; died: median = 3, mode = 1, range = 12; Kruskal–Wallis Chi-square = 7.09, *p* = 0.0078). No important difference in the number of predatory taxa was found between plants where eggs survived and plants where they did not survive (survived: median = 2, mode = 1, range = 7; died: median = 2, mode = 1, range = 7; Kruskal–Wallis Chi-square = 2.44, *p* = 0.1180).

## 4. Discussion

There is tremendous variation reported in the literature regarding monarch egg and larval survival, some of which might depend on methodology [53,54]. This makes it extremely difficult to compare among studies. In an effort to rely only on comparable studies, we restrict our comparisons to studies that used the same protocols; that is, field studies using unrestricted focal individuals. By necessity, we also include comparisons with all studies providing quantitative data on first-generation monarch egg and larval survival. These comparisons are shown in Table 4.

In our study, 13% of monarch eggs survived to the third instar and this varied only slightly between the three years of study. This value is comparable to values reported from several studies conducted in Florida; it is slightly higher than the long-term average recorded by Brower [7], similar to survival reported by Cohen and Brower [55], but lower than the survival observed by Zaluki and Brower [56] (Table 4). On the other hand, our survival was much higher than any of the three studies that included Texas (Table 4). Lynch and Martin [24] found low monarch survivorship in north Texas and northwest Louisiana in the mid-1980s. However, in that study there was extensive variation among sites and, at one site in north Texas, monarchs utilizing *A. viridis* had an estimated survivorship to the third instar of 38% [24]. Some of the variation observed by Lynch and Martin [24] may be due to differences in site characteristics or, alternatively, sample sizes and methodology. The monarch survival that we recorded was also much higher than that recorded in the two other studies conducted in Texas. Calvert found 0% survival to third instar of monarch larvae in a pasture in central Texas in 1995 [23] and an average survival to third instar of 0.24% in three pastures in 1997 and 1998, also in central Texas [25]. As mentioned above, some of these differences could be due to site characteristics. However, there are also methodological considerations that need to be addressed. The first study by Calvert [23] is based on only 61 eggs. It is possible that the small sample size led to an erroneously low estimate of survival. Furthermore, all three of the studies that included Texas are based on counts at single points in time, thereby creating stage-structured data [54,58]. In these studies, survivorship to the third instar is calculated by dividing the number of third instars found by the number of eggs found. There are problems with this approach (e.g., [53,54,59]). First, it does not provide an accurate estimate of the number of eggs that were laid to produce the instars observed during that survey date. This creates a source of error in the survivorship estimate. Second, stage-structured data do not account for how long the third instars detected during the survey had already been alive. As a result, these data do not account for individuals that reached the third instar but subsequently perished prior to the survey date. This omission would inflate estimates of mortality up to the third instar [53]. Lastly, detection probability can be a problem with monarch surveys as young instars are difficult to detect [54,60]. Lack of detection will also inflate estimates of mortality [47,53]. In our study, we followed individuals until they reached the third instar and revisited the plants to ensure that small instars were not simply overlooked or were not temporarily off the host plant. Consequently, our data is less likely to underestimate survival.

The survival rates measured in our study were also considerably higher than survival rates reported for studies using the same focal individual method and applied to later monarch generations further north (Table 4). In our study, the estimated daily survival rate across all three age classes was 0.896, which is much higher than the 0.56 recorded for monarch eggs and first through second instars in Minnesota [33]. In that study, survivorship to the third instar was estimated to be only 1.7%; over seven times lower than the survivorship we measured. In Wisconsin, two studies found survival rates to be less than half of that found in the current study (Table 4) [30,45]. A study in Michigan found that 48 h survival of first instars varied from just over 15% to over 40% depending on disturbance regime [57]. In our study, the equivalent 48 h survival would average 80%. 

Our data indicate that spring-generation monarch survival at our site in Texas was high relative to most other studies with the exception of those conducted in Florida. It is possible that high survivorship is typical of first-generation monarchs in the southern U.S., though a broad geographic analysis based on long-term data suggests otherwise [22]. 

Though much of the analyses of sources of mortality in this study are based on inferences from arthropod occurrence on host plants, there were two known sources of mortality that merit some discussion here. Just over 10% of the mortalities recorded in our study were due to plant damage, a source of mortality that is not well-represented in the literature. Some of this was due to browsing by hares or rabbits as observed in at least one other published account [50]. However, especially in 2018, a large proportion of this plant damage was due to the high winds which are frequent in much of the southern plains in early spring. These winds sometimes, especially for exposed plants, broke the ramets at the base of the plant thereby causing these ramets to wilt and die back. This is a source of monarch egg and larval mortality that does not appear to have been reported elsewhere in the literature. In addition, about 5% of mortalities occurred among eggs that were parasitized by *Trichogramma*. There appears to be almost no research on this source of mortality in North America north of Mexico. In Mexico, *Trichogramma* was an important source of mortality for the eggs of resident populations of monarch butterflies using *Asclepias curassavica* as host plants [61] and in a few cases mortality of monarch butterfly eggs due to *Trichogramma* infection was as high as 100% [62]. Further study on the variation of *Trichogramma* infection among sites and years may be an important area of future study on first generation monarch survival. Collectively, plant damage and egg parasitism accounted for 16% of all the mortalities recorded in our study.

Though our data only provide a snapshot of the arthropod activity on each monarch host plant, the community that was revealed was remarkably rich and diverse. These 77 arthropod taxa occupied the host plants for a variety of reasons. Six taxa were herbivores that either tolerate milkweed plants or are milkweed specialists [63]. Over the course of the study, the host plants were in various stages of flowering. Milkweed flowers produce abundant nectar and, for that reason, milkweed plants attract many different arthropods. In Arizona, *Asclepias tuberosa* flowers are visited by over 80 different species of arthropods [64] and, in Oklahoma, *A. viridis* flowers are visited by over 23 families of insects [65]. Furthermore, milkweed plants like *A. viridis* have a stout growth form that makes the plants attractive to insects seeking physical structures on which to rest or form harborages. Spiders, for example, will select plants based on plant architecture [66] and this seemed to be true of the jumping spiders observed on more than half of the host plants in our study. Many other arthropods are simply transient, using the milkweed plant as a temporary resting place within the larger context of the surrounding plant community. In turn, all of these arthropods attract many different predators to the host plants [33,36]. In our study, 27 of the 77 arthropod taxa observed on monarch host plants were predators and predators represented three of the four most abundant arthropods. 

Our data show that monarch survival was best predicted by the abundance and richness of arthropod taxa that are typically non-predatory. More non-predatory arthropods and a greater number of non-predatory arthropod taxa were associated with greater monarch survival. Despite the fact that predatory arthropods represented three of the most abundant taxa, and that jumping spiders, which are known predators of monarchs [33], were the most frequent arthropods on host plants, neither the abundance nor the number of predatory taxa provided any predictive power in explaining monarch mortality. Our study does not provide information on what caused the observed relationship between increased non-predatory arthropods and increased monarch survival and there are many factors, biotic and abiotic, that might be responsible for covariation between non-predatory arthropod abundance and monarch egg and larval survival. Such factors might include weather conditions, host plant quality, predator dilution, positive indirect top-down effects, and others. We cannot evaluate the effects of weather conditions with the current data, though the three field seasons differed markedly in weather conditions without concordant variation in monarch survival or arthropod abundances. A concurrent study, also conducted on our study site, examined host plant health and found that monarch survival and arthropod diversity and abundance was unrelated to most measures of plant health [67]. Furthermore, our step-wise analysis included measures of host plant quality (number of ramets, number of leaves, length of ramets) but did not select these parameters when alternate parameters in the form of arthropod groups were available. We suggest that two other ecological processes, dilution effects and positive indirect top-down effects, might be important avenues for future research on this system. We provide a brief review of why these effects might be important below. 

The simplest explanation as to why monarch survival increased with higher numbers and diversity of other non-predatory arthropods on the host plant is that the per capita risk of predation may decrease as the number of potential prey items increases, a phenomenon known as the dilution effect [68]. All of the predators identified in our study have been cited as preying on monarch eggs and larvae [31,33,34,36,69] or were observed to do so over the course of our study. They are also all, to varying extents, polyphagous predators. Among polyphagous arthropod predators, diet breadth is often determined by hunting method, encounter rates, infochemical cues, and size [70]. For highly polyphagous predators, the inclusion of a prey item in the diet can be strongly associated with encounter rate [70,71,72,73]. When compared to many of the other potential prey items on the host plants, monarch eggs and monarch larvae up to the third instar are small and solitary. For many of the host plants, there were other larger and more abundant arthropods available as prey, particularly on those plants that were flowering. For this reason, it is unlikely that the predators we observed on the host plants arrived specifically searching for monarch eggs and larvae. Consequently, the consumption of the eggs and larvae, when it occurred, was opportunistic.

Furthermore, even highly polyphagous arthropod predators exhibit prey selectivity such that not all available prey are included in their diets [70]. Monarch eggs and larvae contain cardenolides, whereas most of the other arthropods on the host plant either do not sequester cardenolides or are not as efficient as monarchs in sequestering these compounds [74]. Because some polyphagous predators are averse to prey with high levels of cardenolides [75] they may avoid consuming monarch eggs and larvae in favor of consuming other prey on the host plant. If predators exhibit preferences for other prey items on the plant, then is it possible that such preferences might favor monarch survival above that expected simply on the basis of a dilution effect. This would be a positive, top-down indirect effect [76,77]. Positive top-down indirect effects have been shown to increase herbivore fitness in other invertebrate communities [78]. Specifically, preferential predation by a predator on one prey species can lead to increases in the survival of less preferred prey species [79,80]. Top-down regulation has been proposed as important for monarch survival [28,30,31,33,34,36,69]. However, indirect effects associated with top-down processes are not well documented in monarchs, though they are known to occur. In field and laboratory studies, consumption of monarch larvae by non-native ladybugs (*Harmonia axyridis*), was reduced when aphids (*Aphis nerii*) were present on the host plant [27]. In the current study, higher abundances of other non-predatory arthropods, only a few of which sequester cardenolides, may have favored monarch survival because predators, such as spiders and ants, may have preferentially fed on these other arthropods. This is clearly an area that merits further study.

The role of arthropod biodiversity on monarch egg and larval survival seems to vary extensively among the few studies that have examined it. As found in our study, several studies have found that high biodiversity favors monarch recruitment and survival [22,28,29,38,81]. However, a number of other studies have found that either biodiversity has no influence on survival or it has a negative influence on survival [26,30,33,53]. For example, in Minnesota there was a direct relationship between the presence of spiders and low monarch survival. In that study, the presence of aphids was also associated with lower survival among monarch eggs and larvae [33]. Similarly, in Wisconsin, there was reduced survival when host plants held both ants and aphids [30]. In Michigan, it was found that monarch egg survival was lower in plots with higher plant diversity than it was in plots with low plant diversity [26]. Lastly, in Nebraska it was found that there was little difference in monarch recruitment and survival in urban gardens, where arthropod diversity is expected to be low, and tallgrass prairies, where arthropod diversity would be high [54]. 

We think that some of these differences are due to geography and site characteristics. In our study we focused on the arthropods associated with host plants and we uncovered significant variation among plants. On a larger scale, Lynch and Martin [24] documented considerable variation in monarch survival among study sites in north Texas and northwestern Louisiana. It may be important that the study sites used by Lynch and Martin [24] were all pastures and pastures vary greatly in plant diversity according to how intensively they are managed. Our study area is specifically managed to promote high plant diversity and harbors over 26 species of grasses and over 58 species of forbs (JGK pers. obs.). High plant diversity, in turn, is correlated with high arthropod diversity [82,83]. In addition, it may be important that all of the studies that failed to find a positive influence of arthropod diversity on monarch survival were studies that occurred at higher latitudes. In North America arthropod diversity is higher at low latitudes and lower at high latitudes. Arthropod diversity is particularly high in Texas and Oklahoma [84], where most first generation monarchs originate. For this reason, the influence of arthropod biodiversity on monarch survival might be more important in southern latitudes than in northern latitudes. We suggest that in order for effective monarch conservation to occur, consideration must be given to the influence that functional arthropod communities have on monarch survival in addition to simply adding more milkweed to the landscape. Clearly more research needs to be done on this topic for appropriate and successful conservation strategies to be developed. 

The monarch butterfly was once an abundant species with a continent-wide distribution. It seems likely that the decline of monarch butterflies in North America is tied to the global and serious issue of declining terrestrial arthropods in general [85,86,87,88]. If so, this further emphasizes the need to frame the conservation of monarch butterflies within a broader framework of restoring terrestrial arthropod diversity and the ecological function of the associated arthropod communities. 

## Figures and Tables

**Table 1:** Arthropod taxa associated with 224 eggs used for logistic regression analysis of monarch egg survivorship. Arthropod groups highlighted in yellow are predatory taxa. Percent frequency refers to the percent of monarch eggs or larvae that each taxon was associated with.

Taxon	Common Name	Total Abundance	Frequency	Percent Frequency
Hemiptera, Aphidoidea	Aphid	10,792	80	35.71
Hymenoptera, Formicidae, Others	Other Ants	907	37	16.52
Hymenoptera, Formicidae, *Monomorium minimum*	Little Black Ant	855	74	33.04
Hymenoptera, Formicidae, *Solenopsis invicta*	Red Imported Fire Ant	633	69	30.80
Coleoptera, Curculionidae,	Weevils	471	67	29.91
Arachnida, Acari, Mites	Mites	268	62	27.68
Arthropoda, Others	Other Non-Predatory Arthropods	267	111	49.55
Araneae, Salticidae	Jumping Spiders	247	116	51.79
Arthropoda, Others, Predatory	Other Predatory Arthropods	227	123	54.91
Coleoptera, Chrysomelidae	Other Leaf Beetle	171	65	29.02
Coleoptera, Dermestidae	Dermestid Beetle	139	28	12.50
Diptera	Flies	138	81	36.16
Hemiptera, Cicadomorpha	Leafhopper	137	77	34.38
Hemiptera, Lygaeidae, *Oncopeltus fasciatus*	Large Milkweed Bug	108	38	16.96
Arthropoda, Others	Other Milkweed Herbivores	48	34	15.18
Coleoptera, Others	All Other Beetles	33	22	9.82

**Table 2:** Summary of stepwise logistic regression analysis of survival of monarch eggs or larvae based on arthropod groups (see Table 1) found on host plants. A stepwise selection procedure was used to generate these models with a significance level for entry into the model set at 0.30 and significance level for removal from the model set at 0.35. Models are sorted in order of ascending AICc. Best model is based on minimum corrected AIC Score (AICc), *w_i_* is the Akaike weight of each model.

Model	AICc	_Δ_AICc	*w_i_*	Likelihood Ratio *X*^2^	Model Probability
Other Non-Predatory Arthropods, Mites, Other Ants	251.635	0.000	0.417	16.8877	0.0007
Other Non-predatory Arthropods, Mites	251.724	0.089	0.399	14.7246	0.0006
Other Non-predatory Arthropods	253.290	1.656	0.182	11.1039	0.0009
Intercept Only	262.358	10.723	0.002	-	-
Summary of the best fit model. Concordance of this model was 51.3%.					
Parameter	DF	Estimate	Standard Error	Wald Chi-Square	*p*-value
Intercept	1	−1.4206	0.2028	49.0425	0.0001
Other Non-Predatory Arthropods	1	0.2468	0.0775	10.1330	0.0015
Mites	1	0.0850	0.1133	0.5631	0.4530
Other Ants	1	0.0057	0.00772	0.5456	0.4601

**Table 3:** Summary of stepwise logistic regression analysis of survival of monarch eggs or larvae based on the abundance and richness of predatory and non-predatory arthropods found on host plants. A stepwise selection procedure was used to generate these models with a significance level for entry into the model set at 0.30 and significance level for removal from the model set at 0.35. Models are sorted in order of ascending AICc. The best model is based on minimum corrected AIC Score (AICc), *w_i_* is the Akaike weight of each model.

Model	AICc	_Δ_AICc	*w_i_*	Likelihood Ratio *X*^2^	Model Probability
Number of Non-Predatory Taxa	256.806	0.000	0.508	7.5880	0.0059
Number of Non-Predatory Taxa, Number of Predatory Arthropods	257.003	0.197	0.460	9.4459	0.0089
Intercept Only	262.358	5.552	0.032	-	-
Summary of the best fit model. Concordance of this model was 55.5%.					
Parameter	DF	Estimate	Standard Error	Wald Chi-Square	*p*-value
Intercept	1	−1.5450	0.2586	35.7021	0.0001
Number of Non-Predatory Taxa	1	0.1390	0.0507	7.5190	0.0061

**Table 4:** Comparison of monarch survival measured in previous studies to the survival measured in the current study. All of these are field studies based on eggs and larvae that were not confined to enclosures and which were in outdoor settings presumably exposed to unmanipulated arthropod communities.

Location	Measurement	Value	Equivalent Value in Current Study	Citation
Florida	Survival to 3rd instar	9.2%	13%	Brower et al. 2018 [7]
Florida	Survival to 3rd instar	About 14%	13%	Cohen and Brower 1982 [55]
Florida	Survival to 3rd instar	17–21%	13%	Zaluki and Brower 1992 [56]
Texas and Louisiana	Survival to 3rd instar	3% (0% to 40%)	13%	Lynch and Martin 1993 [24]
Texas	Survival to 3rd instar	0%	13%	Calvert 1996 [23]
Texas	Survival to 3rd instar	0.24%	13%	Calvert 2004 [25]
Minnesota	Daily survival rate, survival to third instar	0.56, 1.7%	0.896, 13%	De Anda and Oberhauser 2015 [33]
Wisconsin	Survival to hatching	35%	63.3%	Borkin 1982 [45]
Wisconsin	Seven-day survival rate	18%	46%	Prysby 2004 [30]
Michigan	48-h survival rate of first instars	15% to 40%	80%	Haan and Landis 2019 [57]

## Data Availability

The data presented in this study are available on request from the corresponding author.

## Appendix A

**Table A1:** Arthropod taxa associated with 224 monarch egg and larva host plants. Rows highlighted in yellow represent predatory taxa. Rows highlighted in green represent herbivorous taxa known to feed on milkweed or observed to do so in this study. All other taxa were considered to be either nectaring: harboring, or transient. Percent frequency is the percentage of eggs that a taxon was associated with.

		Total		Percent
Taxon	Common Name	Abundance	Frequency	Frequency
Hemiptera, Aphidoidea	Aphid	10,792	80	35.71
Hymenoptera, Formicidae, Others	Other Ants	907	37	16.52
Hymenoptera, Formicidae, *Monomorium minimum*	Little Black Ant	855	74	33.04
Hymenoptera, Formicidae, *Solenopsis invicta*	Red Imported Fire Ant	633	69	30.80
Coleoptera, Curculionidae, Baridinae	Flower Weevil	272	40	17.86
Arachnida, Acari, Mites	Mite	268	62	27.68
Araneae, Salticidae	Jumping Spider	246	116	51.79
Coleoptera, Curculionidae, Molytinae	Stem Weevil	167	42	18.75
Coleoptera, Dermestidae	Dermestid Beetle	139	28	12.50
Hemiptera, Cicadomorpha	Leafhopper	137	77	34.38
Coleoptera, Chrysomelidae, Alticini	Flea Beetle	128	48	21.43
Diptera, Unknown	Other Flies	116	72	32.14
Hemiptera, Lygaeidae, *Oncopeltus fasciatus*	Large Milkweed Bug	108	38	16.96
Aranea, Unknown	Other Spider	59	41	18.30
Orthoptera, Caelifera	Grasshopper	50	34	15.18
Coleoptera, Chrysomelidae	Other Leaf Beetle	43	29	12.95
Coleoptera, Unknown	Other Beetles	33	22	9.82
Coleoptera, Curculionidae, Entiminae	Broad-Nosed Weevil	31	20	8.93
Thysanoptera	Thrip	29	18	8.04
Hemiptera, Heteroptera	Other True Bugs	28	24	10.71
Hymenoptera, Apocrita, Unknown Wasps	Wasp	25	20	8.93
Arachnida, Opiliones	Harvestman	24	24	10.71
Araneae, Araneidae	Orb-weaver Spider	22	19	8.48
Diptera, Chironomidae	Midge Fly	22	18	8.04
Hemiptera, Lygaeidae, *Lygaeus kalmii*	Small Milkweed Bug	19	16	7.14
Araneae, Oxyopidae	Lynx Spider	18	17	7.59
Araneae, Thomisidae	Other Crab Spider	18	15	6.70
Hemiptera, Lygaeidae, Unknown	Other Seed Bug	17	9	4.02
Araneae, Lycosidae	Wolf Spider	14	12	5.36
Othoptera, Tettigoniidae	Katydid	13	12	5.36
Myriapoda, Diplopoda	Millipede	13	8	3.57
Collembola	Springtail	12	8	3.57
Diptera, Muscidae	House Fly	11	11	4.91
Araneae, Thomisidae, *Misumena vatia*	Goldenrod Crab Spider	11	8	3.57
Phasmatodea	Stick Insect	10	10	4.46
Coleoptera, Coccinellidae, *Coccinella septempunctata*	Seven-Spotted Ladybeetle	10	10	4.46
Coleoptera, Cerambycidae	Longhorn Beetle	10	7	3.13
Insecta, Unknown Egg	Insect Egg	10	1	0.45
Araneae, Tetragnathidae	Long-jawed Orb Weaver	8	7	3.13
Coleoptera, Coccinellidae, *Harmonia axyridis*	Asian Ladybeetle	8	7	3.13
Diptera, Calyptratae	Other Calyptrate Fly	8	5	2.23
Hymenoptera, Apidae, *Xylocopa* sp.	Carpenter Bee	6	6	2.68
Hemiptera, Reduviidae	Assassin Bug	6	4	1.79
Arachnida, Acari	Tick	5	3	1.34
Hemiptera, Coreidae	Leaf-Footed Bug	5	3	1.34
Othoptera, Grylidae	Field Cricket	4	4	1.79
Coleoptera, Carabidae	Ground Beetle	4	4	1.79
Hymenoptera, Anthophila, Unknown	Other Bee	4	4	1.79
Blattodea, Isoptera	Termite	4	4	1.79
Isopoda	Isopod	4	3	1.34
Neuroptera, Adult	Lacewing	4	3	1.34
Neuroptera, Larvae	Lacewing Larva	4	2	0.89
Araneae, Agelenidae	Grass Spider	3	3	1.34
Hemiptera, Miridae	Plant Bug	3	3	1.34
Coleoptera, Cantharidae	Soldier Beetle	3	3	1.34
Hymenoptera, Apidae, *Bombus* sp.	Bumblebee	3	3	1.34
Hymenoptera, Apidae, *Apis* sp.	Honey Bee	3	2	0.89
Coleoptera, Elateridae	Click Beetle	2	2	0.89
Coleoptera, Tenebrionidae	Darkling Beetle	2	2	0.89
Coleoptera, Staphylinidae	Rove Beetle	2	2	0.89
Hymenoptera, Vespidae	Vespid Wasp	2	2	0.89
Diptera, Sarcophagidae	Flesh Fly	2	2	0.89
Diptera, Tachinidae	Tachinid Fly	2	2	0.89
Diptera, Syrphidae, adult	Flower Fly, adult	2	2	0.89
Coleoptera, Coccinellidae, Larva	Ladybeetle Larva	2	2	0.89
Araneae, Philodromidae	Running Crab Spider	2	2	0.89
Diptera, Tipulidae	Cranefly	2	2	0.89
Hemiptera, Pseudococcidae	Mealybug	2	2	0.89
Hemiptera, Pentatomidae, Asopinae	Predatory Stink Bug	2	1	0.45
Coleoptera, Scarabaeidae	Scarab Beetle	1	1	0.45
Hemiptera, Pentatomoidea	Stink bug, non-predatory	1	1	0.45
Araneae, Salticidae, *Myrmarachne* sp.	Ant-mimic Jumping Spider	1	1	0.45
Lepidoptera, larva	Caterpillar	1	1	0.45
Mecoptera	Scorpion Fly	1	1	0.45
Trichoptera	Caddisfly	1	1	0.45
Lepidoptera, Heterocera	Moth	1	1	0.45
Coleoptera, Curculionoidea, Unknown	Other Weevil	1	1	0.45
